# Persistent Purine Metabolic Abnormality Induces the Aggravation of Visceral Inflammation and Intestinal Microbiota Dysbiosis in Magang Goose

**DOI:** 10.3389/fvets.2021.737160

**Published:** 2021-09-06

**Authors:** Weiqing Ma, Lingjuan Zhou, Yu Li, Daiyang Xia, Jianying Chen, Junpeng Chen, Xianzhi Jiang, Jiangfan Qin, Yujie Zhao, Xiufen Zhang, Heng Wang, Yang Fu, Shanshan Zhu, Huiquan Jiang, Hui Ye, Yongwen Zhu, Zhenping Lin, Wence Wang, Lin Yang

**Affiliations:** ^1^Guangdong Provincial Key Laboratory of Animal Nutrition and Regulation, College of Animal Science, South China Agricultural University, Guangzhou, China; ^2^Shantou Baisha Research Institute of Origin Species of Poultry and Stock, Shantou, China; ^3^Microbiome Research Center, Moon (Guangzhou) Biotech Co., Ltd., Guangzhou, China; ^4^Cofco Feed (Foshan) Co., Ltd., Foshan, China; ^5^Gold Coin Feedmill (Dong Guan) Co., Ltd., Dongguan, China

**Keywords:** purine metabolic, gout, visceral inflammation, intestinal microbiota, Magang goose

## Abstract

Gout is a disease involving abnormal purine metabolism that is widespread in mammals and birds. Goose is especially susceptible for gout in early stage. However, a few studies investigated the ontogenetic pattern of goslings with purine metabolic abnormality. Our studies were conducted to investigate whether persistent purine metabolic abnormality would lead to aggravation of visceral inflammation and intestinal microbiota dysbiosis in goose. A total of 132 1-day-old Magang geese were randomly divided into six replicates and fed a high-calcium and protein meal-based diet from 1 to 28 days. The experiment lasted for 28 days. Liver and kidney damages were observed in 14- and 28-day-old Magang geese, and liver inflammation increased with increasing age. In 28-day-old Magang geese, serum CAT and liver GSH-Px activity were significantly reduced. Furthermore, jejunum intestinal barrier was impaired and the abundance of *Bacteroides* was significantly reduced at the genus level. Collectively, the high-calcium and high-protein (HCP) meal-based diet caused liver and kidney damage in 28-day-old Magang geese, leading to hyperuricemia and gout symptoms, and the intestinal barrier is impaired and the intestinal flora is disrupted.

## Introduction

Gout is one of the most common metabolic diseases in mammals and birds, which is a form of inflammatory arthritis in the setting of hyperuricemia (1). The incidence of gout is rising worldwide, and in 2018, the prevalence of gout was ~3% in humans, with a tendency for a disproportionately higher prevalence in the younger generation, because of improvements in living standards and changes in dietary habits (2). Due to the lack of urate oxidase and glutamine synthetase in poultry, ammonia was difficult to convert to urea through the guanine cycle and was hardly excreted to the outside of the body. Therefore, hyperuricemia and gout easily occurred in poultry, especially in goslings.

Gosling gout involves multiple mechanisms such as increased uric acid synthesis and decreased metabolism caused by abnormal purine metabolism and astrovirus infection (3, 4). There are two types of gout in poultry: one is articular gout and the other is visceral gout (5, 6). Articular gout mainly leads to the deposition of monosodium urate (MSU) microcrystals in and around joints, due to the disruption of purine metabolism and a reduction in uric acid excretion (7, 8). Visceral gout tends to occur in poultry, especially in goose from age 7 to 15 days. Goslings with gout showed enlarged kidneys and urate crystals (5). The goose industry in China experienced a large-scale outbreak of gout in 2016, and the mortality rate in geese was up to 50% (9, 10).

Numerous studies have indicated that a high-calcium and high-protein (HCP) diet would lead to hyperuricemia and gout in poultry, because of the high uric acid level in the body and impaired kidney function (11). However, few studies investigated the ontogenetic pattern of goslings with purine metabolic abnormality treated by a persistent HCP diet. Therefore, the present study was conducted to investigate whether persistent purine metabolic abnormality would lead to aggravation of visceral inflammation and intestinal microbiota dysbiosis in goose. Our results may provide new insights for the prevention of gout and dietary therapy in poultry and mammals.

## Materials and Methods

### Animals and Treatment

All animal experiments were conducted in accordance with the Guangdong Provincial Laboratory Animal Welfare and Ethical Review Guidelines and were approved by the Animal Welfare Committee of South China Agricultural University. A total of 132 Magang geese (half male and half female) were obtained from Hongxing Hatchery (Qingyuan, Guangdong, China) and randomly divided into six replicates of 22 geese each. All geese were housed in a net bed with a heat lamp for the first 7 days of the experiment. The temperature during the first 7 days was 32 ± 1°C and reduced by 2.5 ± 0.5°C per week to a final temperature of 26°C. At day 14 and 28, geese were weighed and sacrificed for sample collection. Over the entire experimental period (1–28 days), the geese were allowed *ad libitum* consumption of feed and water. We sought to make a gout model by feeding geese a high-calcium (3.78%) and high-protein (24.25%) diet from 1 to 14 days. In order to prevent excessive mortality, we changed a persistent secondary HCP diet (Ca 1.98% and CP 20.10%) from 14 to 28 days after gout phenotype appeared. According to the National Research Council (12, 13) standards, geese requirement is 0.65% calcium and 20% protein in diets during the early stage. We produce a high-calcium and high-protein diet based on calcium and protein of 0.65% and 18% (14). Other nutrients conformed to the recommendations of the National Research Council (1994). The composition and nutritional level of the diet are shown in Table 1.

**Table 1:** The compositions of basic diet and the nutritional level (air-dried basis, %).

**Item**	**Days 1–14**	**Days 15–28**
**Ingredient**		
Corn	52.45	61.40
Corn albumen power	29.46	20.40
Fishmeal	1.52	1.52
Wheat bran	4.80	10.00
Limestone	8.94	3.90
Calcium phosphate	1.50	1.50
Lysine	0.50	0.50
Solid methionine	0.16	0.16
Minerals premix^a^	0.09	0.09
Vitamin premix^b^	0.025	0.025
Choline chloride	0.14	0.14
Sodium chloride	0.30	0.30
Bentonite	0.115	0.065
Total	100	100
**Nutritional level**		
ME (MJ/kg)	11.42	11.76
Crude protein	24.25	20.10
Crude fiber	1.57	1.82
Ca	3.78	1.98
AP	0.50	0.50
Lys	0.71	0.70
Met	0.55	0.48

a *Vitamin premix provided the following per kilogram of diet: vitamin A, 40,000,000 IU; vitamin D_3_, 10,000,000 IU; vitamin E, 100,000 mg; vitamin K_3_, 20,000 mg; vitamin B_1_, 10,000 mg; vitamin B_2_, 30,000 mg; vitamin B_6_, 20,000 mg; vitamin B_12_, 12,100 mg; biotin, 500 g; pantothenic acid, 60,000 mg; folic acid, 5,000 mg; nicotinic acid, 200,000 mg; ethoxyquin, 500 mg.*

b *The mineral premix provided the following per kg of diet: Cu, 8–12 g; Fe, 100–110 g; Mn, 120–130 g; Co, 0.3–0.5 g; Se, 0.3–0.5 g; I, 0.7–0.9 g; moisture, ≤ 3%*.

### Data and Sample Collection

All geese were weighed at the beginning and end of the experiment, and the number of dead geese was recorded, and this information was used to calculate the following values:

$$\begin{array}{l} \text{Average daily gain }\left(\text{ADG}\right)\;\left(\text{g}/\text{day}\right)\;=\text{ Increase in body}\\ \text{ weight during the trial}\\ \text{ period }\left(\text{g}\right)/\text{day }\left(\text{d}\right)\\ \text{ Organ index }\left(\text{g}/\text{kg}\right)\;=\text{ Organ weight }\left(\text{g}\right)/\\ \text{ Body weight }\left(\text{kg}\right)\\ \text{ Mortality rate }=\text{ Number of deaths geese}/\\ \text{ Total geese }\times \;100\text{\%} \end{array}$$

On day 14 and 28, 12 geese (two from each pen) were randomly selected and euthanized by cervical dislocation. Although sex is not the main factor affecting gosling gout, we still take it into account (15). Each replicate has half male and female numbers of geese in our experiment in order to eliminate the impact of gender on treatment. Serum, cecal chyme, and tissue samples were collected in sterile cryogenic vials, snap-frozen in liquid nitrogen, and stored at −80°C until further processing.

### Quantitative Real-Time PCR

Total RNA was isolated and analyzed by quantitative RT-PCR. The PrimerScript RT reagent kit with gDNA Eraser (Takara, Japan) was used for reverse transcription. DNASTAR Lasergene 7.1 software was used to design the upstream and downstream primers of the liver: phosphoribosyl pyrophosphate aminotransferase (*PPAT*), adenosine deaminase (*ADA*), xanthine oxidase (*XOD*), organic anion-transporting polypeptide 1A1 (*OATP1A1*), kidney xanthine oxidase (*XOD*), organic anion-transporting 1 (*OAT1*), glucose transporter 9 (*GLUT9*), and Urat 1 (*URAT1*) and the internal reference gene β-actin. The primers were synthesized by Shanghai Shenggong Biological Engineering Co., Ltd. The primer sequence is shown in Table 2. The Premix Taq kit was used to perform gradient PCR to verify primers and explore annealing temperature. SYBR Green Quantitative PCR kit (Takara, Japan) was used to perform fluorescent quantitative PCR on Applied Biosystems 7500 (Thermo Fisher, USA) real-time fluorescent quantitative PCR instrument. The relative expression of the target gene was calculated by the 2^−Δ*ΔCt*^ method.

**Table 2:** Gene primer sequence information.

**Genes**	**Primer sequences (5^**′**^-3^**′**^)**	**GenBank accession number**	**Product length (bp)**
*β-actin*	F:TCCTGCGGCAT CCACGAGA	NM_0013101421.1	199
	R:CCGCCGATCCAGA CCGAGTA		
*PPAT*	F:GGCCAGGAGAGTG CTGGAAT	NM_001004401.1	122
	R:CATACAGCTTCT TCAGGCTG		
*ADA*	F:ACCTCGTAAATC AGGGACTG	XM_027472902.1	148
	R:TGGCCACCACAGA GTTGTTT		
*XOD*	F:ACTGTCGAAGG CATAGGA	NM_205127.1	415
	R:GCTGGAACTCG GAAGAAT		
*OATP1A1*	F:GTCCTTGCTGACT GCAACAC	XR_003956207.1	143
	R:TGAAACACCATG TTGGTTCC		
*OAT1*	F:CTGCATCTTCCT GTACACTG	XM_032283060.1	155
	R:CGTAGATGAAGA GAGGCATG		
*GLUT9*	F:TGGCAGGTCATTACTG TGGTTGTC	XM_013099415.3	197
	R:CGTCCGAGCCGC TCAATAACTAAG		
*URAT1*	F:GGCTTCACCTT CTACGGCCT	XM_032415038.1	116
	R:AGCAGCAGGGTG CCGATCTT		

### Biochemical Marker Analyses

Serum calcium, phosphorus, alkaline phosphatase (ALP), gamma-glutamyl transpeptidase (GGT), alanine aminotransferase (ALT), and aspartic transaminase (AST) were detected by a fully automatic biochemical analyzer (AU480; Beckman Coulter, Inc., Brea, USA). Other serum biochemical indexes, uric acid (UA), creatinine (Cr), blood urea nitrogen (BUN), XOD, antioxidant indexes, LPS, and inflammation cytokine levels were measured using enzymatic kits from Nanjing Jiancheng Bioengineering Institute, Nanjing, China.

### Kidney, Liver, and Jejunum Histology Analysis

Kidney, liver, and jejunum cross-sections were fixed in 10% buffered formalin, and 5-μm paraffin-embedded sections were stained with hematoxylin and eosin (H&E). The slides were examined under light microscopy by a pathologist in a blinded manner. The areas in tissues were evaluated using Leica QWin software (Leica Microsystems, Wetzlar, Germany).

### Microbial Diversity Analysis

#### DNA Extraction and PCR Amplification

Microbial DNA was extracted from cecal chyme samples using the E.Z.N.A.® soil DNA Kit (Omega Bio-Tek, Norcross, GA, USA) according to the protocols of the manufacturer. The final DNA concentration and purification were determined by a NanoDrop 2000 UV–vis spectrophotometer (Thermo Scientific, Wilmington, DE, USA), and DNA quality was checked by 1% agarose gel electrophoresis. The V3–V4 hypervariable regions of the bacterial 16S rRNA gene were amplified with primers 338F (5′-ACTCCTACGGGAGGCAGCAG-3′) and 806R (5′-GGACTACHVGGGTWTCTAAT-3′) by a thermocycler PCR system (GeneAmp 9700, Applied Biosystems, Foster City, CA, USA). The PCR reactions were conducted using the following program: 3 min of denaturation at 95°C, 27 cycles of 30 s at 95°C, 30 s of annealing at 55°C, 45 s of elongation at 72°C, and a final extension of 10 min at 72°C. PCR reactions were performed in a triplicate 20-μl mixture containing 4 μl of 5 × FastPfu buffer, 2 μl of 2.5 mM dNTPs, 0.8 μl of each primer (5 μM), 0.4 μl of FastPfu polymerase, and 10 ng of template DNA. The resulting PCR products were extracted from 2% agarose gel and further purified using the AxyPrep DNA Gel Extraction Kit (Axygen Biosciences, Union City, CA, USA) and quantified using QuantiFluor™-ST (Promega, Madison, WI, USA) according to the protocols of the manufacturer.

#### Illumina MiSeq Sequencing

Purified amplicons were pooled in equimolar amounts and paired-end sequenced (2 × 300) on an Illumina MiSeq platform (Illumina, San Diego, CA, USA) according to the standard protocols of Majorbio Bio-Pharm Technology Co., Ltd. (Shanghai, China).

#### Processing of Sequencing Data

Raw fastq files were quality-filtered by Trimmomatic and merged by FLASH with the following criteria: (i) the reads were truncated at any site with an average quality score <20 over a 50-bp sliding window. (ii) Sequences that overlapped by more than 10 bp were merged according to their overlap with a mismatch of no more than 2 bp. (iii) Sequences of each sample were separated according to barcodes (exact matching) and primers (allowing two nucleotide mismatches), and reads containing ambiguous bases were removed. Operational taxonomic units (OTUs) were clustered with a 97% similarity cutoff using UPARSE (version 7.1, http://drive5.com/uparse/) with a novel “greedy” algorithm that performs chimera filtering and OTU clustering simultaneously. The taxonomy of each 16S rRNA gene sequence was analyzed by the RDP Classifier algorithm (http://rdp.cme.msu.edu/) against the Silva (SSU123) 16S rRNA database using a confidence threshold of 70%.

### Statistical Analysis

All results are presented as mean ± standard error (SEM). Student's *t*-test was used to independently compare day 14 HCP group with day 28 HCP group. All statistical analyses were conducted using SAS software (version 9.2, Raleigh, NC, USA). A value of *p* < 0.05 was used as the criterion for statistical significance.

## Results

### Hepatic and Nephritic Inflammatory Response to Persistent HCP Diet

**Figure 1:**
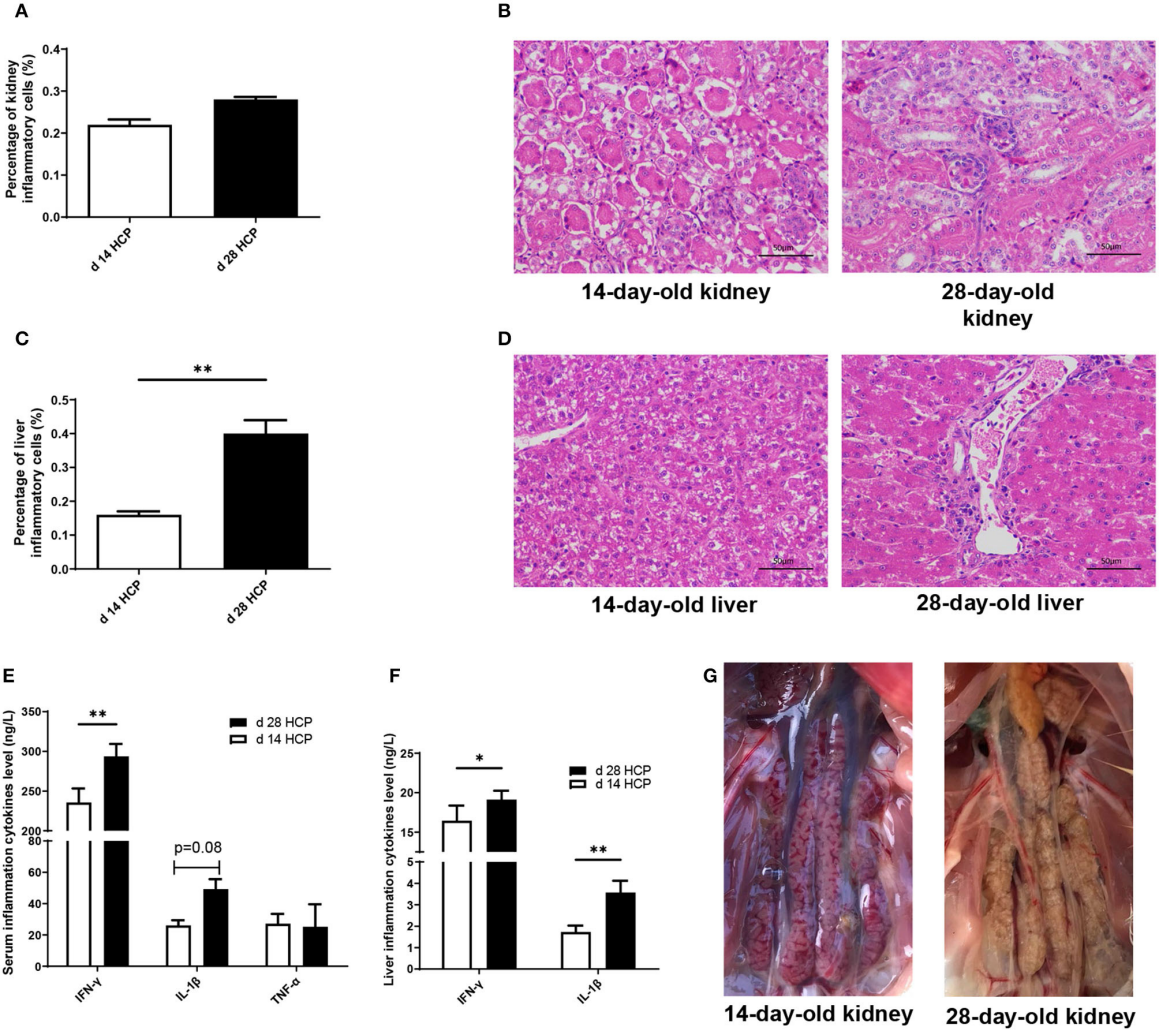
A high-calcium and high-protein (HCP) meal-based diet causes an inflammatory response in geese. **(A)** Percentage of inflammatory cells in the kidney. **(B)** Kidney of day 14 and 28 HCP (bar = 50 μm); the image was taken at magnification of ×400. **(C)** Percentage of inflammatory cells in the liver. **(D)** Liver of day 14 and 28 HCP (bar = 50 μm); the image was taken at magnification of ×400. **(E)** Serum inflammation cytokine level. **(F)** Liver inflammation cytokine level. **(G)** Clinical signs of kidney appearance in the development of gout (bar = 1 cm). Error bars represent the standard error of the mean (*n* = 6/group). *Mean significant difference (*p* < 0.05). **Mean extremely significant difference (*p* < 0.01).

Growth performance of Magang geese on day 14 and 28 is shown in Supplementary Table 1. Although there was no significant difference in ADG, Magang geese increased in mortality rate by 20.31% from day 14 to 28 (Supplementary Table 1). The HCP meal-based diet had little effects on organ indexes, except the kidney (Supplementary Figure 1). No statistically significant differences were observed in the percentage of kidney inflammatory cells between the day 14 HCP and the day 28 HCP groups (Figure 1A). The percentage of kidney inflammatory cells was 25% higher on day 14 than that on day 28, and the percentage of inflammatory cells in liver on day 28 was significantly higher than that on day 14 (*p* < 0.01; Figure 1C). Kidney and liver on day 14 and 28 showed inflammatory cell infiltration and hemorrhage (Figures 1B,D). Compared with those in the day 14 HCP group, the serum IFN-γ and liver IL-1β levels in the day 28 HCP group were greatly increased (*p* < 0.01; Figures 1E,F). The liver IFN-γ level in the day 28 HCP group was significantly higher than that in the day 14 HCP group (*p* < 0.05; Figure 1F). There was no significant difference in the serum TNF-α level (*p* > 0.05; Figure 1E). A common feature observed in both groups was that the kidneys exhibited predominant gross lesions, which were mottled and swollen, and there was visceral urate deposition on the serous surfaces of the kidney on day 28, which is typical of severe kidney damage in gout geese (Figure 1G).

### Serum Biochemical Indexes

**Figure 2:**
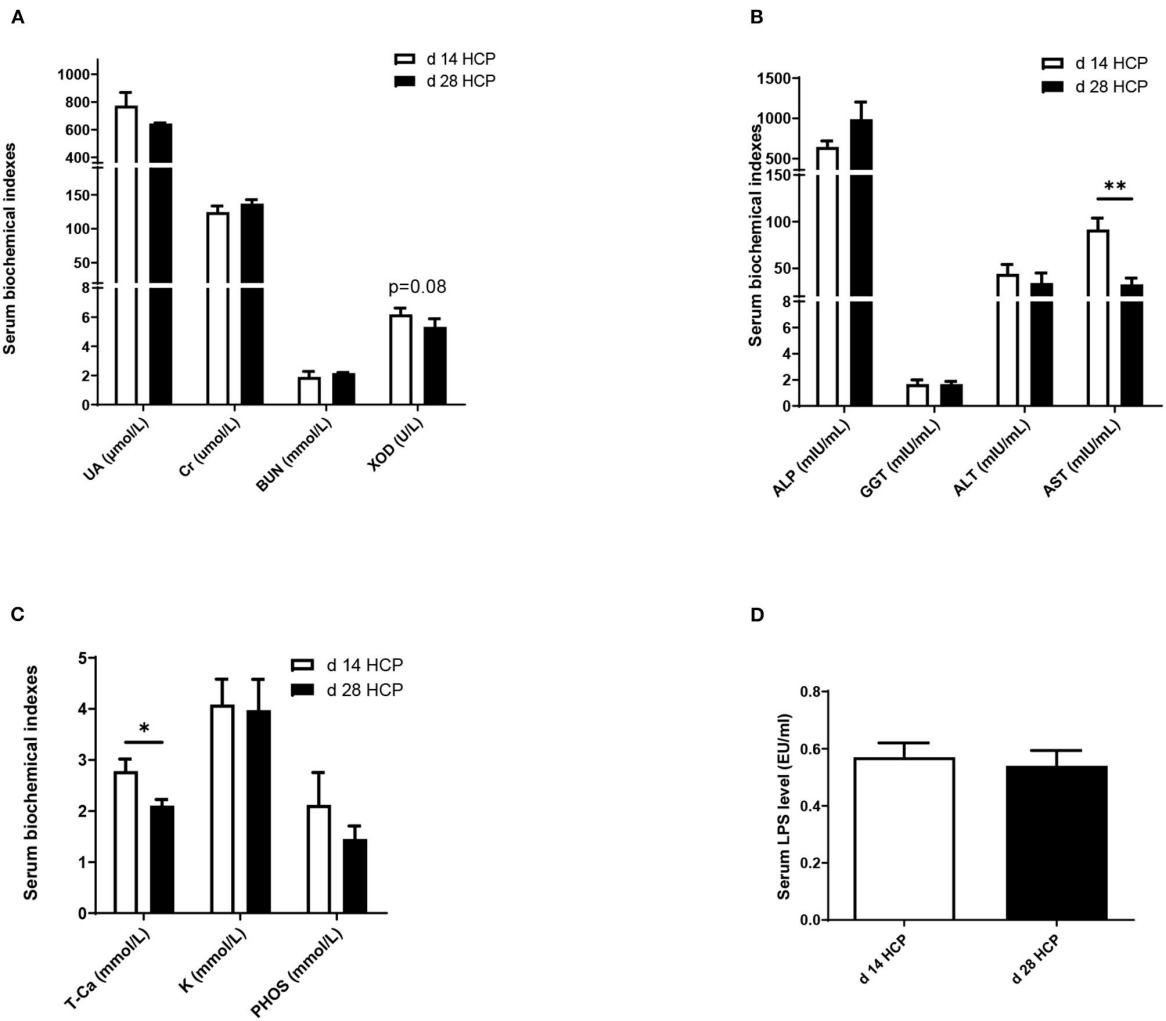
Effect of HCP meal-based diet on serum biochemical indexes of Magang geese on day 14 and 28. **(A–C)** Serum biochemical indicators. **(D)** Serum LPS level. *Mean significant difference (*p* < 0.05) and **mean extremely significant difference (*p* < 0.01).

There were no significant differences in serum UA, Cr, or BUN between the two groups. The XOD level in the day 28 HCP group was 25% less than that in the day 14 HCP group, but no significant differences were observed between the two groups (*p* = 0.08; Figure 2A). The serum AST level in the day 28 HCP group was much lower than that in the day 14 HCP group (*p* < 0.01; Figure 2B). The serum T-Ca level in the day 28 HCP group was significantly lower than that in the day 14 HCP group (*p* < 0.05; Figure 2C). There was no significant difference in the serum LPS level (*p* > 0.05; Figure 2D).

### Serum and Liver Antioxidant Indexes

**Figure 3:**
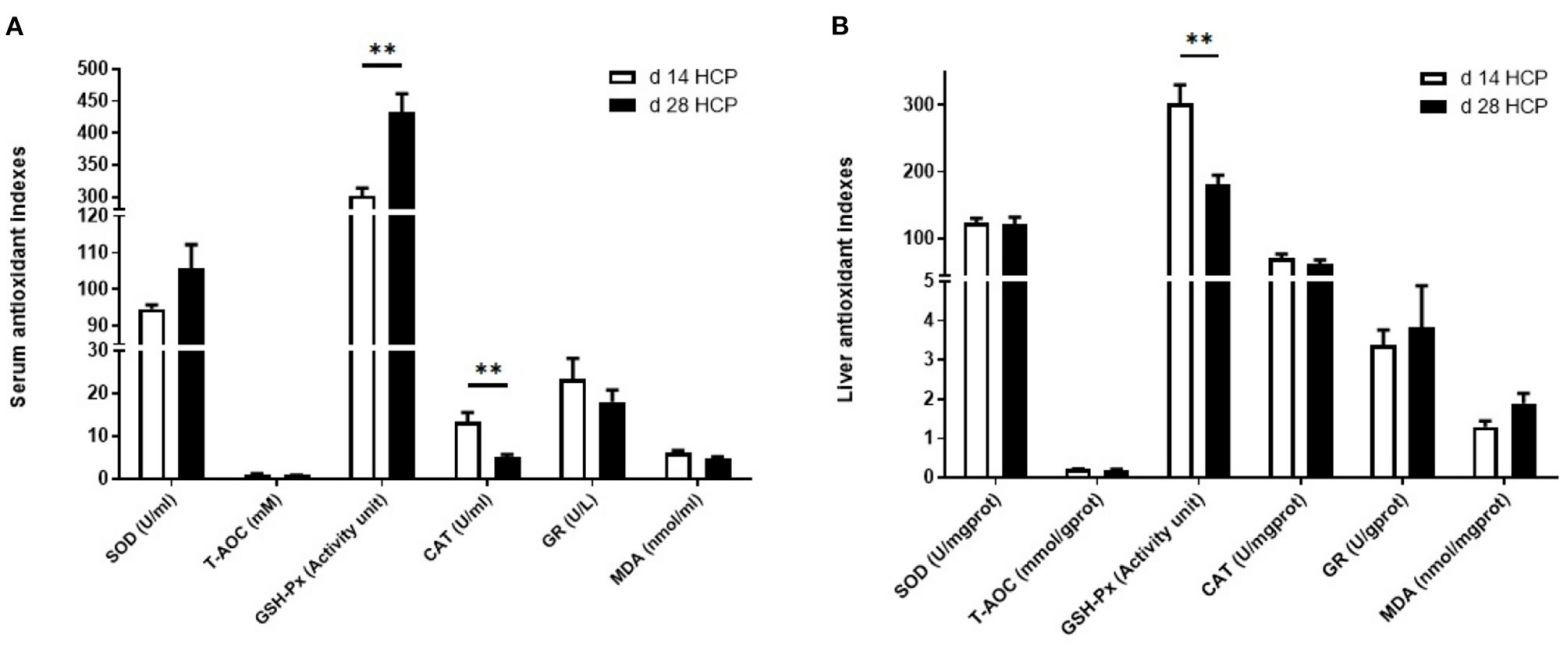
Effect of HCP meal-based diet on serum and liver antioxidant indexes of Magang geese on day 14 and 28. **(A)** Serum antioxidant indicators. **(B)** Liver antioxidant indicators. **Mean extremely significant difference (*p* < 0.01).

The serum GSH-Px level in the day 28 HCP group was much higher than that in the day 14 HCP group (*p* < 0.01). The serum CAT level in the day 28 HCP group was significantly lower than that in the day 14 HCP group (*p* < 0.01; Figure 3A). The liver GSH-Px level in the day 28 HCP group was significantly lower than that in the day 14 HCP group (*p* < 0.01; Figure 3B).

### Relative Gene Expression for the Purine Metabolic Pathway and Uric Acid Transporter

**Figure 4:**
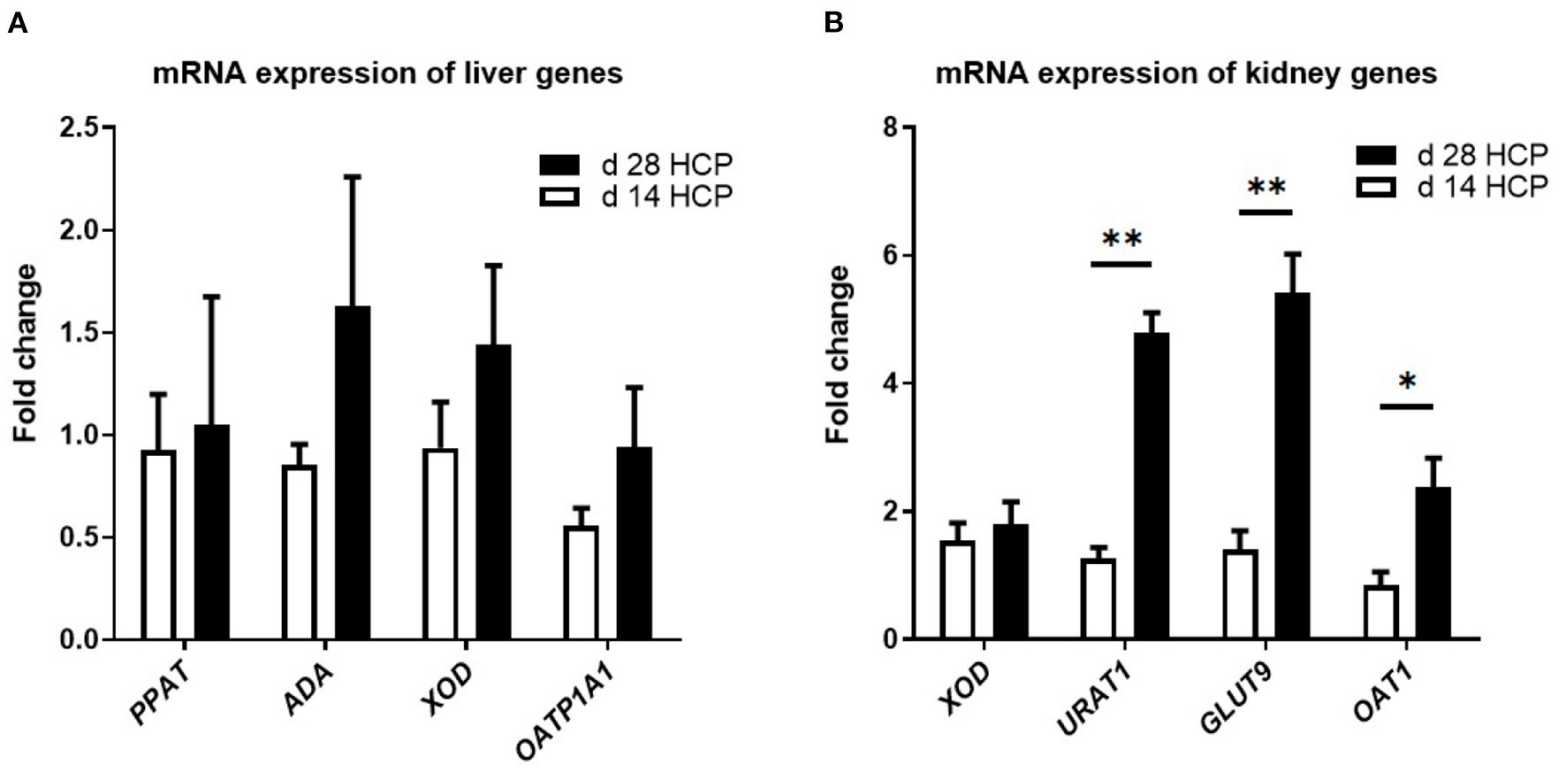
Effect of HCP meal-based diet on the relative expression of genes related to uric acid metabolism pathway in the liver and uric acid transporter in the kidney of Magang geese on day 14 and 28. **(A)** *ADA, PPAT, XOD*, and *OATP1A1* mRNA relative expression. **(B)** *XOD, URAT1, GLUT9*, and *OAT1* mRNA relative expression. β-Actin was used as an internal control to normalize target gene transcript levels. *Mean significant difference (*p* < 0.05) and **mean extremely significant difference (*p* < 0.01).

There were no significant differences in the expression levels of the *OATP1A1, PPAT, ADA*, and *XOD* genes, which are involved in uric acid metabolism, between the two groups (*p* > 0.05; Figure 4A). There was no significant difference in the expression of the *XOD* gene in the kidney, which is involved in uric acid metabolism, between the two groups (*p* > 0.05; Figure 4B). The expression levels of *URAT1* and *GLUT9* in the day 28 HCP group were much higher than those in the day 14 HCP group (*p* < 0.01), and the expression level of *OAT1* in the day 28 HCP group was significantly higher than that in the day 14 HCP group (*p* < 0.05; Figure 4B).

### Jejunal Morphology Observation

**Figure 5:**
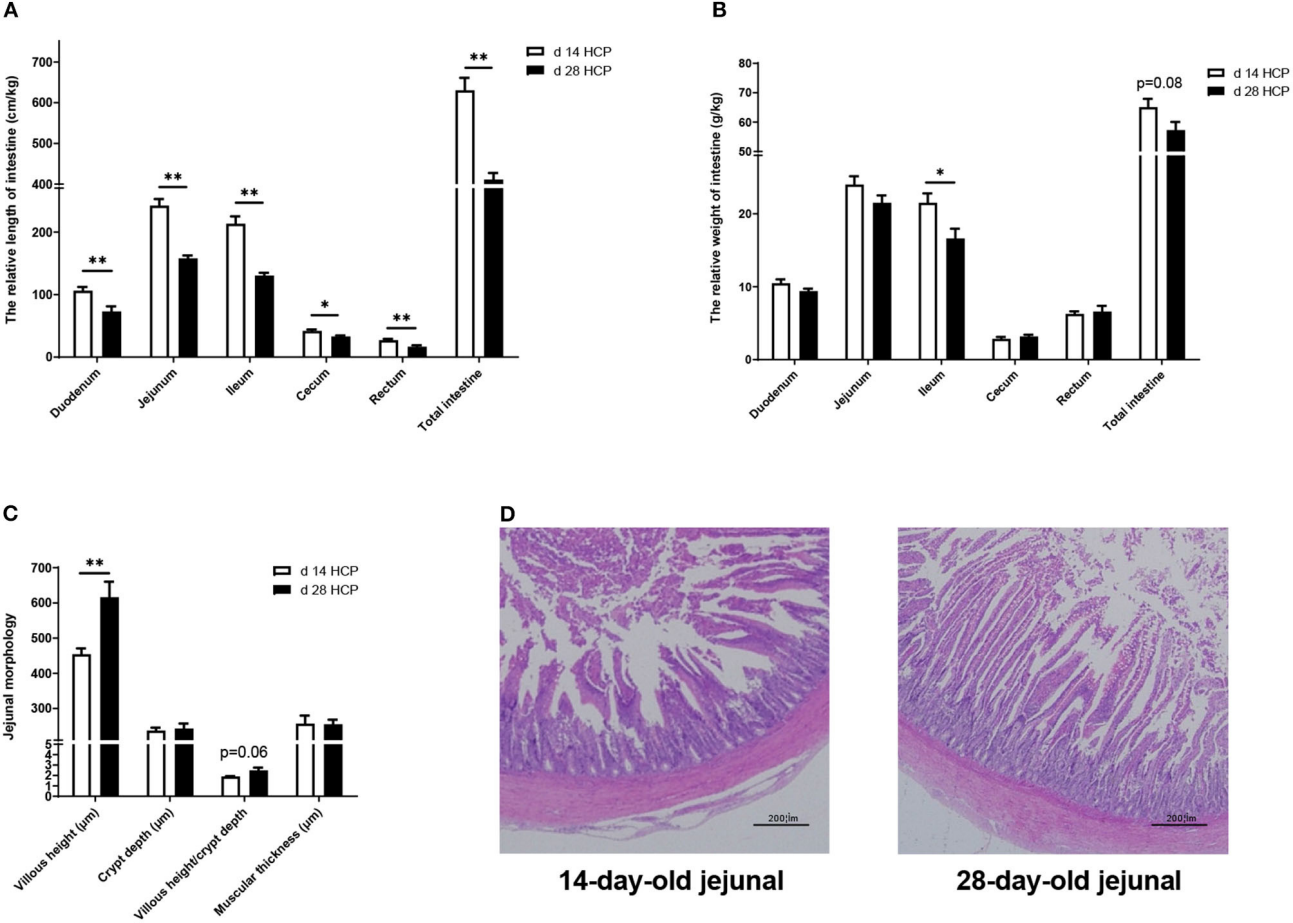
Effect of HPC meal-based diet on gut development of Magang geese on d 14 and d 28. **(A)** The relative length of intestine. **(B)** The relative weight of intestine. **(C)** Jejunal morphology. **(D)** Jejunal morphology of d 14 and d 28 HCP. *mean significant difference (p < 0.05) and **mean extremely significant difference (p < 0.01). The image was taken at magnification of 20×, scale bar, 200μm.

The relative length of the duodenum, jejunum, ileum, rectum, and total intestine of geese in the day 28 HCP group was significantly lower than that in the day 14 HCP group (*p* < 0.01; Figure 5A). The relative length of the cecum and the relative weight of the ileum of geese in the day 28 HCP group were significantly lower than those in the day 14 HCP group (*p* < 0.05; Figures 5A,B). Compared with 14-day-old Magang geese, 28-day-old Magang geese villus height was extremely significantly increased, and villus height/crypt depth increased by 23.29%, but not significantly (*p* = 0.06; Figure 5C). At the age of 14 days, the jejunum villus of geese had a disordered arrangement, the intestinal gland was sparse, and intestinal epithelial cells were not clear in outline and irregularly arranged (Figure 5D). At the age of 28 days, the jejunum villus of the Magang geese was significantly damaged, crypt depth increased, jejunal epithelium shed apparently, and the lamina propria was exposed (Figure 5D).

### Intestinal Microbiota Dysbiosis of Goose With Gout

**Figure 6:**
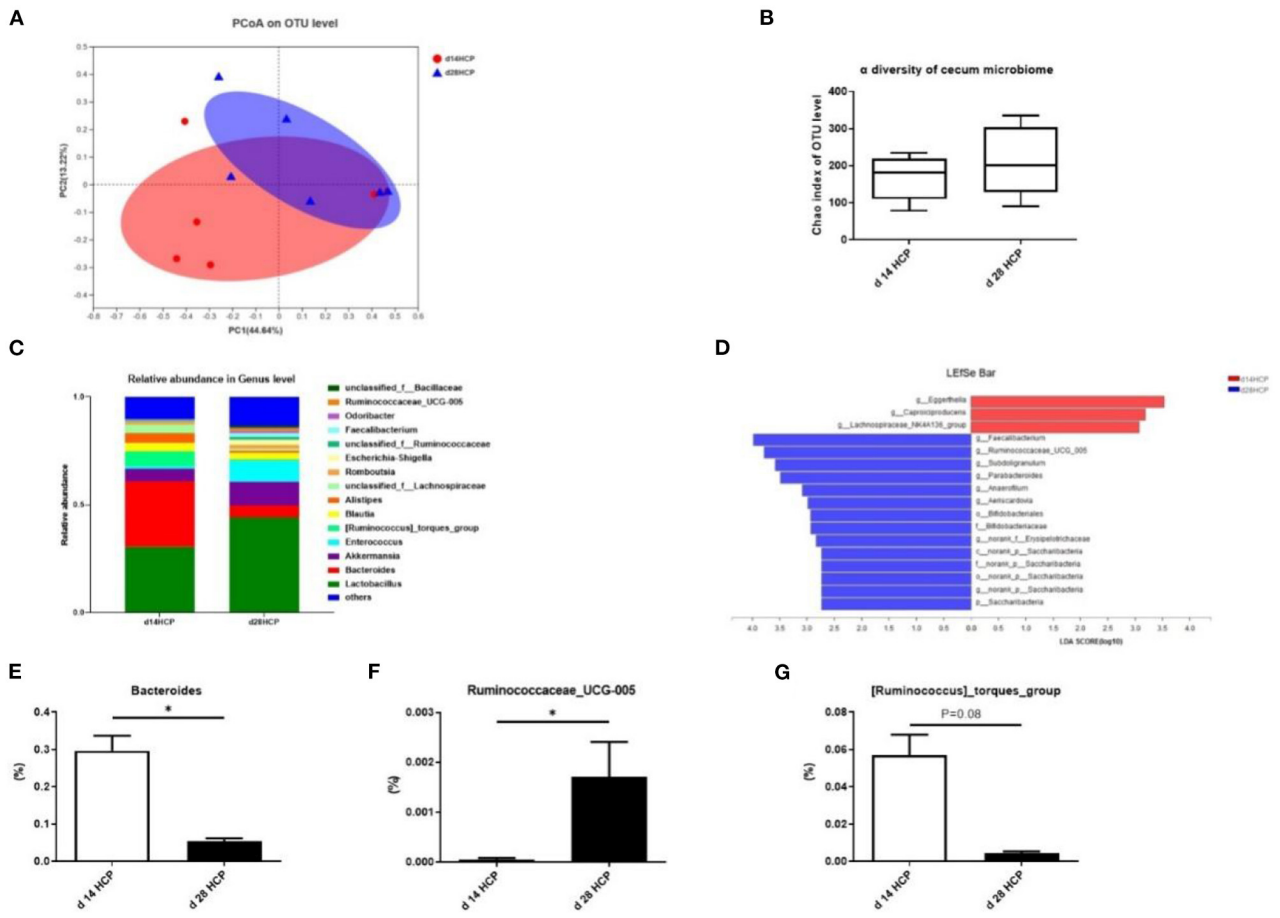
Effect of HCP meal-based diet on cecum microbial diversity of Magang geese on day 14 and 28. **(A)** Principal component analysis of microbial communities in the cecum from the day 14 HCP group and day 28 HCP group. **(B)** Alpha diversity: Chao index. **(C)** Relative abundance in genus. **(D)** LEfSe identified the most differential under the influence of the HCP meal-based diet. The red bars represent the species with high abundances in the day 14 HCP group, and the green bars represent the species with high abundances in the day 28 HCP groups. The higher the LDA score, the more significant difference of abundance between groups. **(E–G)** The abundance of *Bacteroides, Ruminococcaceae_UCG_005*, and [*Ruminococcus*]*_torques_group* between the day 14 HCP group and the day 28 HCP group. Data are expressed as mean ± SEM (*n* = 6/group). Error bars represent the standard error of the mean (*n* = 6/group). *Mean significant difference (*p* < 0.05).

As the age of the day changes, there was no significant difference in total observed species and cecal microbial diversity (Supplementary Figures 2A,B). The abundance of *Eggerthella* dropped by a big margin, and the abundance of *Akkermansia, Enterococcus*, and *Faecalibacterium* has risen sharply (Supplementary Figures 2C–F). The abundance of *Lactobacillus* and *Lactobacillus_aviarius* increased slightly (Supplementary Figures 2G,H). At the phylum and species levels, there was a relative abundance of cecal microbes in goose (Supplementary Figures 2I,J). PCoA analysis of the intestinal microbiota at day 14 and 28 showed that the microbial community was affected (Figure 6A). No significant differences were observed in cecal microbial diversity between the day 14 HCP and day 28 HCP groups (Figure 6B). Analysis of the biological abundance of cecal microbes showed that the dominant flora of the cecum of Magang geese were *Firmicutes* and *Bacteroides* on day 14 and these populations were reduced on day 28. At the genus level, *Enterococcus* and *Akkermansia* were increased, and [*Ruminococcus*]*_torques_group* and *Alistipes* were decreased. At the species level, *Bacteroides fragilis* and *Bacteroides barnesiae* were decreased (Figure 6C). To further analyze the effect of an HCP meal-based diet on the composition of the cecal microflora, we compared the abundance of the two groups of microflora and identified the different main microflora through LEfSe analysis: *Eggerthella, Faecalibacterium*, and *Ruminococcaceae_UCG_005* (Figure 6D). Compared with that in the day 14 HCP group, the abundance of *Bacteroides* in the cecum of the day 28 HCP group was significantly decreased (*p* < 0.05; Figure 6E), the abundance of *Ruminococcaceae_UCG_005* was significantly increased (*p* < 0.05; Figure 6F), and the abundance of [*Ruminococcus*]*_torques_group* tended to be decreased (*p* = 0.08; Figure 6G).

## Discussion

In this study, our results showed that continuous HCP diet would cause persistent purine abnormality in Magang geese, which is accompanied by aggravated inflammation of the liver and kidneys. With increasing age, the activities of liver antioxidants, such as GSH-Px, were decreased significantly. In addition, we also found that the expression of proteins related to urate transport, such as *URAT1, GLUT9*, and *OAT1*, was significantly increased. It is worth noting that the gut microbial composition of 28-day-old Magang geese has changed. At the genus level, the abundance of *Bacteroides* was significantly reduced, and the abundance of *Ruminococcaceae_UCG_005* was significantly increased.

Visceral gout more commonly occurs in poultry farming, which would induce hyperuricemia and the accumulation of urates in the kidney, heart, liver, peritoneum, etc. (16). Previous studies also reported that a high-protein and high-calcium diet could induce severe hypouricemia and gout in poultry (6). The levels of CP and Ca in gosling diet were approximately around 16% CP and 1% Ca in conventional production (5, 17–19). In our experiment, we used high-calcium (3.78%) and high-protein (24.25%) diets to induce hypouricemia in goslings from day 1 to 14. In order to prevent the high mortality in geese, we changed the diet to a persistent secondary HCP diet (Ca 1.98% and CP 20.10%) after the gout phenotype appeared. In addition, this paper does not involve comparison with the control group, because there are many previous studies on gout induced by normal feed and high-calcium and high-protein feed, and our focus is on the longitudinal dynamic study of the ontogeny of Magang geese with continuous high calcium and high protein, especially in the gosling stage of 14 and 28 days. We want to find out the pathogenesis and new solutions of gout from the dynamic changes of the body in the process of gout.

Studies have shown that gout caused by urate was mediated by the TLR4/NF-κB signaling pathway, and the IL-1β released was linearly related to it (20, 21). Destruction of the intestinal barrier induced the translocation of gut-derived LPS from the gut to the liver, which increases the expression of inflammatory factors such as IL-1β and TNF-α in the liver (22, 23). Previous studies showed that the serum LPS level was <0.2 EU/ml in normal poultry (24). In our results, serum LPS levels in geese from day 14 to 28 were maintained at higher levels (>0.54 EU/ml). Both IFN-γ and IL-1β in serum and liver of the day 28 group were significantly higher than those in the day 14 group, which means that a persistent HCP diet aggravated liver inflammation in geese. At the same time, we also observed inflammation in the liver of the day 28 HCP group through the section.

A high-calcium diet led to kidney damage, especially extensive denaturation and necrosis of poultry renal tubules (25). A previous study showed that a high-protein diet could cause serious damage to the kidneys of the goslings, the renal tubules and ureters were swollen, and the epithelial cells showed hydropic degeneration (26). In our experiment, the percentages of nephric inflammation cells maintained high levels from day 14 to 28, and the value in the day 28 group was 27.27% higher than that in the day 14 group. H&E staining showed severe renal tubular epithelial cell necrosis, vacuolar degeneration, and exfoliation with increasing age.

Geese with hyperuricemia have a higher blood uric acid level compared with basal diet geese, approximately more than 400 μmol/L (27). In our results, serum UA concentrations in geese were more than 600 μmol/L from day 1 to 28, which demonstrated that geese were suffering severe gout. An HCP meal-based diet caused kidney damage in goslings, mainly manifested by increased serum uric acid, creatinine, urea nitrogen content, and XOD activity (28). Studies have shown that high protein in the diet could promote arginine synthesis, thereby increasing the serum creatinine content (29, 30). Furthermore, high-calcium diets could increase blood calcium levels, and a high-calcium level in serum tends to accelerate the transformation of xanthine dehydrogenase into xanthine oxidase, which produces more uric acid, nitric oxide (NO), and reactive oxygen radicals, thereby exacerbating renal damage (5). In this experiment, compared with the day 14 group, the serum AST level in the day 28 group was significantly decreased and the expression of *URAT1* in the kidney was increased, which means that geese showed enhanced self-healing with age.

High protein can promote the synthesis of purines and pyrimidines, this process needs to consume a lot of ATP, and the production of ATP will lead to the accumulation of ROS, which leads to changes in redox state of the body. Excessive ROS can also cause the destruction of mitochondrial membrane, resulting in the release of cytochrome C, thus activating the caspase-9 apoptosis pathway. Under normal circumstances, the body will self-repair the subhealth state, including the redox system, autophagy, and apoptosis (31, 32). Feeding chickens with 5% calcium or 25% crude protein diet, blood NO, CAT, and GST significantly increased, and an increase in the level of antioxidant enzymes indicates that the body has a compensatory antioxidant response, which may be triggered by the damage of high-protein diet to other organs (33–35). Our results showed that the serum GSH-Px level in day 28 geese was significantly increased, while the serum CAT level and liver GSH-Px level were significantly decreased, which may be due to the compensatory enhancement of the antioxidant capacity of other organs caused by the HCP diet.

Current evidence shows that gout is associated with comorbidities, such as type 2 diabetes and metabolic syndrome (36). Many enzymes are involved in the purine metabolic pathway, and dysfunction of these enzymes increases uric acid production and blocking of renal tubules, leading to severe hyperuricemia (37). In our experiment, the gene expressions of *XOD, ADA, PPAT*, and *OATP1A1* in the day 28 group were slightly higher than those in the day 14 group, although there were no significant differences between the two groups. Recent studies on the pathogenesis of avian gout indicated that the main factor in avian gout is the mechanism of the uric acid excretion barrier. These two kinds of transporters, URAT1 and GLUT9, constitute the main pathway of urate reabsorption in the kidney, and nearly all urate is reabsorbed by the proximal tubule (38). The expression of *OAT1* in renal tissue was reversibly regulated by hyperuricemia and was accompanied by changes in organic ion transport (39). In this study, with increasing age, the expressions of *URAT1, GLUT9*, and *OAT1* in the kidney were significantly increased with a persistent HCP diet, which indicated that nephric urate accumulation aggravated renal injury. On the other hand, the higher expression of renal transporters also demonstrated that geese have a compensatory mechanism to protect against external damage.

The intestinal barrier is the first line of defense against foreign antigens. Intestinal barrier damage would result in the translocation of bacteria from the intestine to bloodstream liver or other distant organs (40). The studies of Xi et al. have shown that gout induced intestinal barrier dysfunction and intestinal permeability increased (15). In this study, the abnormal purine metabolism caused by the HCP diet changes the shape of the jejunum; compared with the day 14 HCP group, the HCP diet has more obvious intestinal damage to 28-day-old Magang geese, which is mainly manifested in the obvious shedding of jejunal epithelium and exposure of lamina propria, and intestinal mucosal damage was further aggravated.

Cecal microorganisms are affected by diet and age. Under normal circumstances, there is a balance system of intestinal flora, that is, the species and quantity of probiotics and harmful bacteria fluctuate within a certain range (41). When there is disease or stress, harmful bacteria multiply in large numbers. The fecal microbiome and metabolome have also been shown to reflect the presence of gout (4). As indicators of gout in the fecal microbiome, some intestinal bacteria would participate in the metabolism of purines and UA, such as *Escherichia coli, Lactobacillus*, and *Pseudomonas* (42–44). Gout in goslings mainly occurs around the age of 10–15 days, when the composition of the digestive tract flora in goslings is not yet stable (45, 46). Experiments have demonstrated the depletion of *pseudo Bifidobacteria*, and the enrichment of *Ruminococcaceae* in gout may cause severe inflammatory responses (15, 47). In our study, a persistent HCP diet significantly increased the abundance of *Ruminococcaceae_UCG_005* and decreased the abundance of *Bacteroides* in goose cecum. At the phylum level, the *Firmicutes/Bacteroidetes* ratio in the day 28 HCP group was greatly increased compared with that in the day 14 HCP group. Many studies have shown that the *Firmicutes/Bacteroidetes* ratio is related to lipid metabolism and amino acid metabolism, and the *Firmicutes/Bacteroidetes* ratio increased significantly in obese people (48, 49). *Faecalibacterium prausnitzii*, which has anti-inflammatory properties, has been reported to contribute to intestinal health through butyric acid production and could be heavily depleted in gout patients (50, 51). In our results, *Faecalibacterium* appeared at day 28, perhaps because the autoimmune response of the body slightly reduced inflammation after intestinal injury, leading to the spontaneous recovery of intestinal immunity.

In a word, this study found that continuous HCP diets reduced the abundance of *Bacteroides* and increased the abundance of *Ruminococcaceae_UCG_005* in the cecum of Magang geese. The changes of these two bacteria may be related to abnormal purine metabolism, which can be used as in-depth research on potential target flora for the treatment of gout.

## Conclusions

A persistent HCP diet can cause aggravated inflammation and morphological damage in the liver and kidney of 14- to 28-day-old Magang geese. With increasing age, the serum redox balance is disrupted and the liver antioxidant capacity is continuously decreased. The expression of urate transporters *URAT1, GLUT9*, and *OAT1* increased significantly. The intestinal barrier is impaired and the intestinal flora is disrupted, with a decrease in the abundance of *Bacteroides* and an increase in the abundance of *Ruminococcaceae_UCG_005*.

## Data Availability Statement

The datasets presented in this study can be found in online repositories. The names of the repository/repositories and accession number(s) can be found below: NCBI; SUB9958184.

## Ethics Statement

The animal study was reviewed and approved by Animal Ethics Committee of South China Agricultural University. Written informed consent was obtained from the owners for the participation of their animals in this study.

## Author Contributions

WM and WW carried out the main experiments and wrote the manuscript. ZL, LY, and DX designed the experiments. LZ, JiC, XJ, JQ, and YZha provided materials and technical support. XZ, HW, YF, SZ, and HJ helped with the data analysis. HY and YZhu provided assistance for sample analysis. YL provided funding and participate in the revision of the final manuscript. All authors contributed to the article and approved the submitted version.

## Conflict of Interest

XJ was employed by Microbiome Research Center, Moon (Guangzhou) Biotech Co., Ltd., JQ was employed by Cofco Feed (Foshan) Co., Ltd., YZha was employed by Gold Coin Feedmill (Dong Guan) Co., Ltd. The remaining authors declare that the research was conducted in the absence of any commercial or financial relationships that could be construed as a potential conflict of interest.

## Publisher's Note

All claims expressed in this article are solely those of the authors and do not necessarily represent those of their affiliated organizations, or those of the publisher, the editors and the reviewers. Any product that may be evaluated in this article, or claim that may be made by its manufacturer, is not guaranteed or endorsed by the publisher.
