# Supplementary material for: Persistent Purine Metabolic Abnormality Induces the Aggravation of Visceral Inflammation and Intestinal Microbiota Dysbiosis in Magang Goose
Source: Front Vet Sci. 2021 Sep 6;8:737160. doi: 10.3389/fvets.2021.737160 (PMC8452157; doi:10.3389/fvets.2021.737160)
Supplement: Supplementary file 1 [file Data_Sheet_1.pdf]

Table S1. Effect of HPC meal-based diet on growth performance from 14 to 28 days of Magang geese

Item	d 14 HCP	d 28 HCP
Weight (g)	231.60±4.50	484.00±16.01
Average daily gain (**ADG**) (g/d)	16.56±0.71	18.02±2.69
Mortality rate %	18.56	22.33

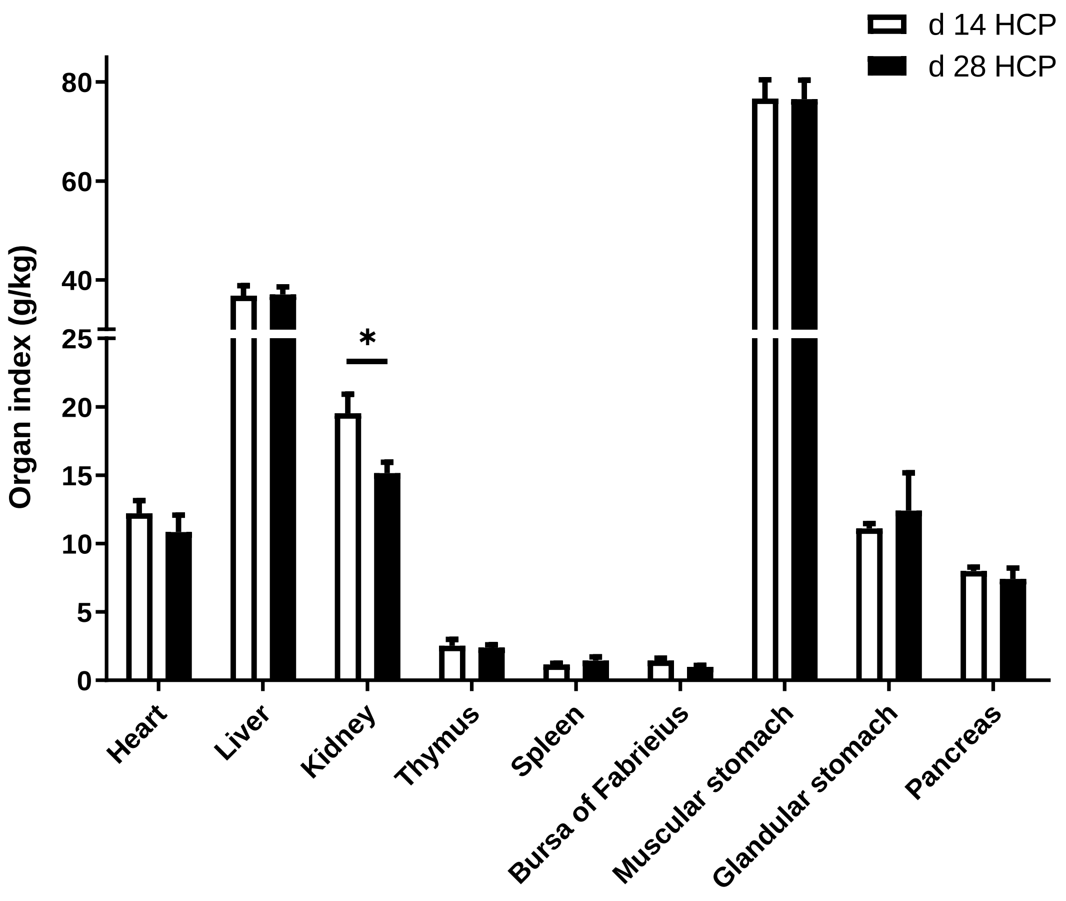

Figure S1. Effect of HPC meal-based diet on organ indexes from 14 to 28 days of Magang geese. * mean significant difference ($p<0.05$).

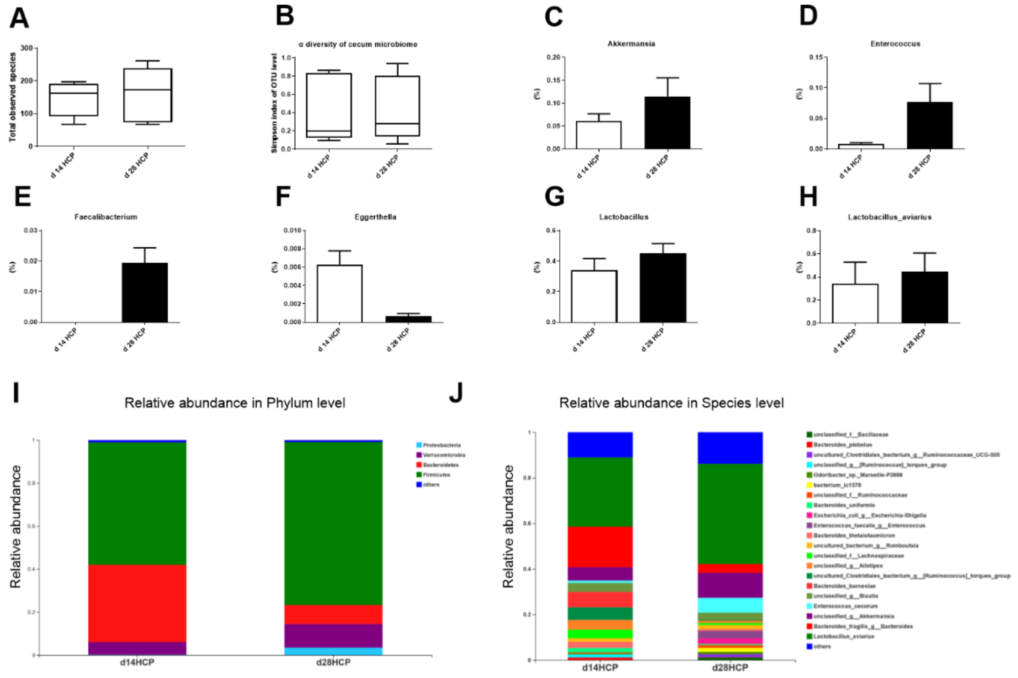

Figure S2. Effect of HPC meal-based diet on cecum microbial diversity of Magang geese on d 14 and d 28. (**A**) Total observed species. (**B**) Alpha diversity: Simpson index. (**C-H**) Comparisons among groups on the relative abundance of the most differential *Akkermansia*, *Enterococcus*, *Faecalibacterium*, *Eggerthella*, *Lactobacillus* and *Lactobacillus_aviarius*. Error bars represent the standard error of the mean. (n = 6/group). (**I**) Relative abundance of bacteria at Phylum level. OTUs with an occurrence lower than 1% are not represented. (**J**) Relative abundance of Species in each group.
